# Circulating Syndecan-1 as a Predictor of Persistent Thrombocytopenia and Lethal Outcome: A Population Study of Patients With Suspected Sepsis Requiring Intensive Care

**DOI:** 10.3389/fcvm.2021.730553

**Published:** 2021-09-07

**Authors:** Kosaku Hatanaka, Takashi Ito, Yutaro Madokoro, Chinatsu Kamikokuryo, Shuhei Niiyama, Shingo Yamada, Ikuro Maruyama, Yasuyuki Kakihana

**Affiliations:** ^1^Department of Emergency and Intensive Care Medicine, Kagoshima University Graduate School of Medical and Dental Sciences, Kagoshima, Japan; ^2^Department of Systems Biology in Thromboregulation, Kagoshima University Graduate School of Medical and Dental Sciences, Kagoshima, Japan; ^3^R&D Center, Shino-Test Corporation, Sagamihara, Japan

**Keywords:** sepsis, glycocalyx, syndecan 1, thrombocytopenia, endotheliopathy

## Abstract

**Background:** Sepsis is defined as life-threatening organ dysfunction caused by dysregulated host responses to infection. Recent studies have suggested that endotheliopathy may be the common basis for multiple organ failure in sepsis. Under septic conditions, accumulation of proteases accelerates shedding of proteoglycans, such as syndecan-1, from the endothelial surface, resulting in augmented leukocyte adhesion to the vascular wall, enhanced vascular permeability, and intravascular coagulation. The purpose of this study was to determine the potential utility of syndecan-1 as a biomarker linking endotheliopathy to organ failure.

**Methods:** One hundred patients with suspected infections who were admitted to the intensive care unit (ICU) at Kagoshima University Hospital were consecutively enrolled in the study. Serum syndecan-1 levels were measured using an in-house enzyme-linked immunosorbent assay. The difference between serum syndecan-1 levels in 28-day survivors and non-survivors was analyzed by the Mann–Whitney *U*-test. Receiver-operating characteristics curve analysis with area under the curve calculation was used to quantify the predictive performance of serum syndecan-1 for 28-day mortality. The correlations between serum syndecan-1 and coagulation markers were analyzed by Spearman's rank correlation test.

**Results:** Serum syndecan-1 levels in non-survivors were significantly higher than those in survivors on Day 1 and Day 3 (*P* < 0.01). Among multiple organ failures, coagulation failure and renal failure were significantly correlated with serum syndecan-1. Spearman's rank correlation test indicated that serum syndecan-1 was weakly but significantly correlated with disseminated intravascular coagulation score (rho = 0.33, *P* < 0.01). Patients with serum syndecan-1 ≥21.4 ng/mL showed delayed recovery from thrombocytopenia relative to patients with serum syndecan-1 <21.4 ng/mL.

**Conclusions:** Elevated circulating syndecan-1 on the first day of ICU admission was associated with persistent thrombocytopenia and lethal outcome in patients with suspected sepsis.

## Introduction

Sepsis is a leading cause of death among critically ill patients in non-coronary intensive care units (ICUs). It is defined as life-threatening organ dysfunction caused by dysregulated host responses to infection (1). Assessment of organ dysfunction is the cornerstone for clinical diagnosis of sepsis, and can be accomplished by use of the sequential organ failure assessment (SOFA) score. Recent studies have suggested that endotheliopathy may be the common basis for multiple organ failure in sepsis (2–4). For example, endothelial injury under septic conditions results in augmented leukocyte adhesion to the vascular wall, enhanced vascular permeability, and intravascular coagulation, all of which exacerbate inflammatory and hypoxic organ failure.

**Figure 1:**
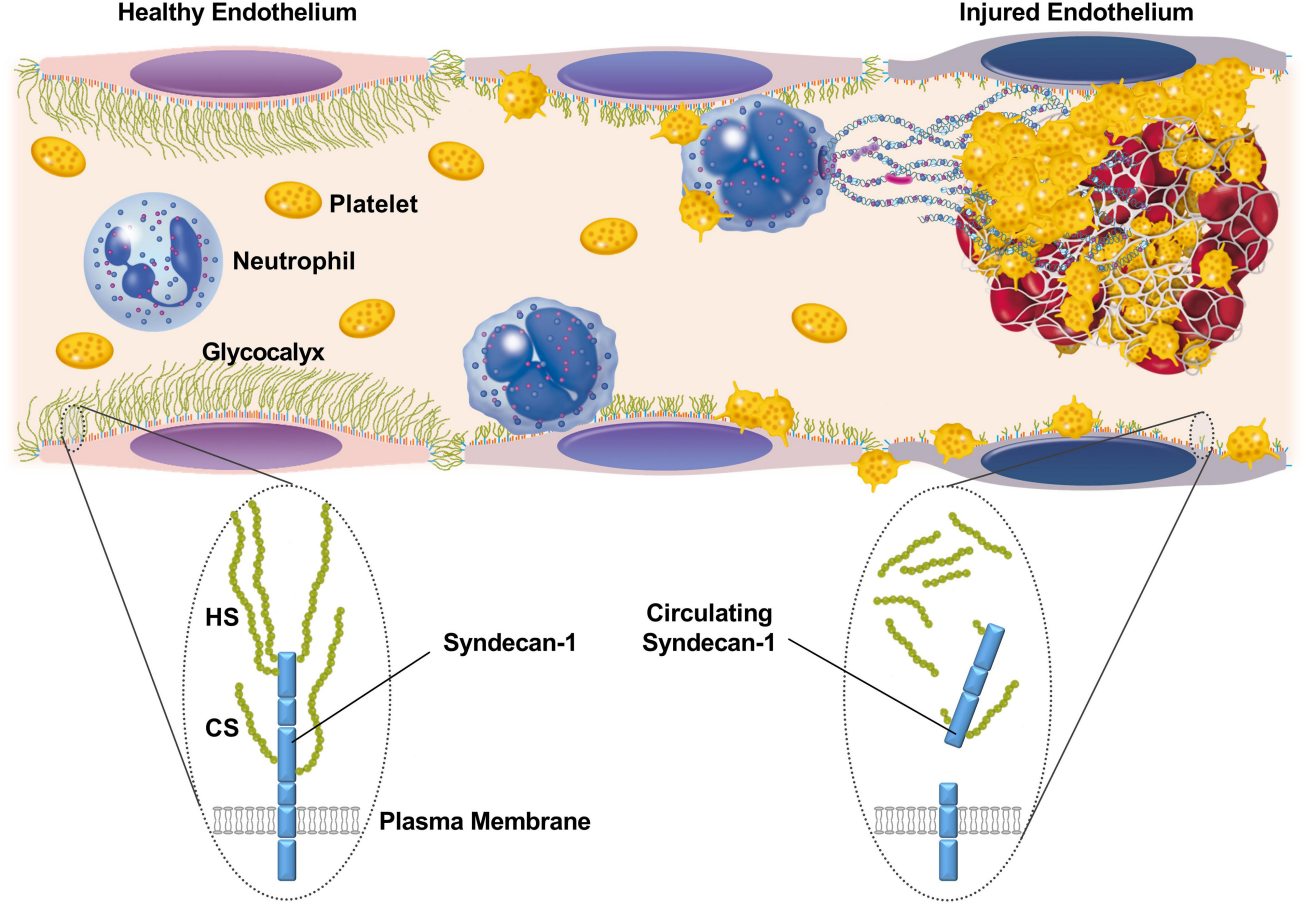
Schematic representation of the glycocalyx and its component, syndecan-1, on the surface of endothelial cells. The luminal surface of endothelial cells is normally coated with a glycocalyx that acts as a barrier against the adhesion of circulating cells or leakage of plasma components. Syndecan-1 is one of the major core proteins to which glycosaminoglycans, such as heparan sulfate (HS) and chondroitin sulfate (CS), become covalently attached. During sepsis, the glycocalyx becomes thinner and sparser, partly through the enzymatic degradation of proteoglycans and glycosaminoglycans. This results in augmented leukocyte adhesion to the vascular wall, enhanced vascular permeability, and intravascular coagulation.

The luminal surface of endothelial cells is normally coated with a glycocalyx composed of membrane-bound proteoglycans, glycosaminoglycan chains, glycoproteins, and adherent plasma proteins (5). The endothelial glycocalyx is highly hydrated, and acts as a barrier against the adhesion of circulating cells and/or leakage of plasma components. During sepsis, however, the glycocalyx becomes thinner and sparser (6), partly through degradation of glycosaminoglycans and proteoglycans by enzymes such as heparanase, hyaluronidase, and metalloproteinases (7). This disruption of the glycocalyx results in augmented leukocyte adhesion to the vascular wall, enhanced vascular permeability, and intravascular coagulation (Figure 1).

Syndecan-1 is one of the major core proteins to which glycosaminoglycan chains become covalently attached (5). Under inflammatory conditions, accumulation of proteases, including matrix metalloproteinases, thrombin, and plasmin, accelerates shedding of syndecan-1 from the endothelial surface (8, 9). As a consequence, circulating syndecan-1 levels can become increased in patients with sepsis, especially in those with multiple organ failure (10). Recent studies have suggested that circulating syndecan-1 levels may be associated with coagulation failure and disseminated intravascular coagulation (DIC) (11, 12). These findings suggest the potential utility of syndecan-1 as a biomarker linking endotheliopathy to organ failure. However, the relationships between circulating syndecan-1 and dynamic changes in platelet responses to injured vascular walls under septic conditions remain unclear. In the present study, by analyzing a cohort of patients with suspected sepsis, we show that elevated circulating syndecan-1 may be associated with refractory thrombocytopenia during the first week of ICU stay.

## Methods

### Study Population

This was a single-center observational study approved by the Ethics Committee of Kagoshima University Graduate School of Medical and Dental Sciences, Kagoshima, Japan (210026). Written informed consent was obtained from all patients for research use of their leftover blood samples after routine blood tests. The sample size was calculated using the n.wilcox.ord program in R version 4.0.2 with statistical power of 0.8, alpha error of 0.05, and fraction of non-survivors of 0.2. The expected proportions of non-survivors with very low, low, high, and very high serum syndecan-1 (categorized by the quartiles for survivors) were 0.12, 0.12, 0.12, and 0.64, respectively, as determined by reference to a data set from a previous study (13). Based on the sample size estimation, we enrolled 100 consecutive patients with suspected infections who were admitted to the ICU at Kagoshima University Hospital between June 2018 and November 2019. Serum samples were obtained in 99 patients within 24 h after ICU admission (Day 1) and in 96 patients on Day 3. After anonymization, these samples were used for measurements of serum syndecan-1 levels. Clinical data, including SOFA score, Acute Physiology and Chronic Health Evaluation II (APACHE II) score, and Systemic Inflammatory Response Syndrome (SIRS) score were also anonymized and analyzed. DIC was diagnosed in accordance with the criteria established by the Japanese Association for Acute Medicine (JAAM) (14) and the International Society on Thrombosis and Hemostasis (ISTH) (15).

### Measurement of Serum Syndecan-1 Levels

Polystyrene microtiter plates (Nunc, Roskilde, Denmark) were coated with 100 μL/well of anti-syndecan-1 polyclonal antibody (R&D Systems, Minneapolis, MN, USA) in phosphate-buffered saline (PBS), and incubated overnight at 37°C. After three washes with PBS containing 0.05% Tween-20, the remaining binding sites were blocked by incubation with 400 μL/well of PBS containing 1% bovine serum albumin (BSA) for 2 h. The plates were washed again, and incubated with 100 μL/well of diluted standard (recombinant syndecan-1, R&D Systems) and plasma samples (1:10 dilution in 0.2 mol/L Tris pH 7.4, 0.15 mol/L NaCl, and 1% BSA) for 24 h at room temperature. After washing, the plates were incubated with 100 μL/well of peroxidase-conjugated anti-syndecan-1 monoclonal antibody (R&D Systems) for 2 h at room temperature. The plates were washed again, and incubated with the chromogenic substrate 3,3′,5,5′-tetramethylbenzidine (Dojindo Laboratories, Kumamoto, Japan) for 30 min at room temperature. The reaction was terminated by addition of 0.35 mol/L Na_2_SO_4_, and the absorbance at 450 nm was measured with a 680 microplate reader (Bio-Rad, Hercules, CA, USA). The lower detection limit of the assay was 0.5 ng/mL, and linearity was observed in the range up to 50 ng/mL. The intra-assay and inter-assay coefficients of variation were 4.5–7.1% and 3.1–9.5%, respectively, at 12.5–25 ng/mL.

### Measurement of Coagulation Markers

Plasma fibrin(ogen) degradation products (FDP) and prothrombin time (PT) were determined using LPIA FDP (LSI Medience, Tokyo, Japan) and HemosIL RecombiPlasTin (Instrumentation Laboratory Company, Bedford, MA, USA), respectively, according to the manufacturers' instructions. Platelet count was determined using an XE-5000 automated counting device (Sysmex Corporation, Kobe, Japan).

### Statistical Analyses

Serum syndecan-1 levels are shown as median (lower quartile–upper quartile). Statistical analyses were performed using SPSS version 26 (IBM Inc., Armonk, NY, USA). Serum syndecan-1 levels in 28-day survivors were compared with those in non-survivors by the Mann–Whitney *U*-test. Receiver-operating characteristics (ROC) curve analysis with area under the curve (AUC) calculation was used to quantify the predictive performance of serum syndecan-1 for 28-day mortality. The relationships between serum syndecan-1 and coagulation markers were analyzed by Spearman's rank correlation test. A *P*-value of < 0.05 was considered statistically significant.

## Results

### Baseline Characteristics of the Patients

The baseline characteristics of the 100 patients are shown in Table 1. All 100 patients had a SOFA score of ≥2, 94 patients were SIRS-positive, 55 patients were DIC-positive (JAAM criteria), and 24 patients had overt DIC (ISTH criteria) on Day 1 of ICU admission. Nineteen patients had died by Day 28.

**Table 1:** Basic characteristics of the study patients.

	**All**	**Survivors**	**Non-survivors**	***P***
	***n* = 100**	***n* = 81**	***n* = 19**	
Age	72 (25–91)	72 (25–91)	73 (35–89)	0.316
APACHE II	21 (8–47)	19 (8–43)	33 (23–47)	<0.01
SOFA score	10 (2–21)	9 (2–18)	15 (7–21)	<0.01
SIRS score	3 (0–4)	3 (0–4)	3 (1–4)	0.48
DIC score (JAAM)	4 (0–8)	4 (0–8)	6 (1–8)	<0.01
DIC score (ISTH)	3 (0–8)	3 (0–6)	4 (2–8)	<0.01

*Median (minimum-maximum) values are shown*.

*Differences between survivors and non-survivors were analyzed by the Mann-Whitney U-test*.

### Circulating Syndecan-1 Is Associated With Fatal Outcome

**Figure 2:**
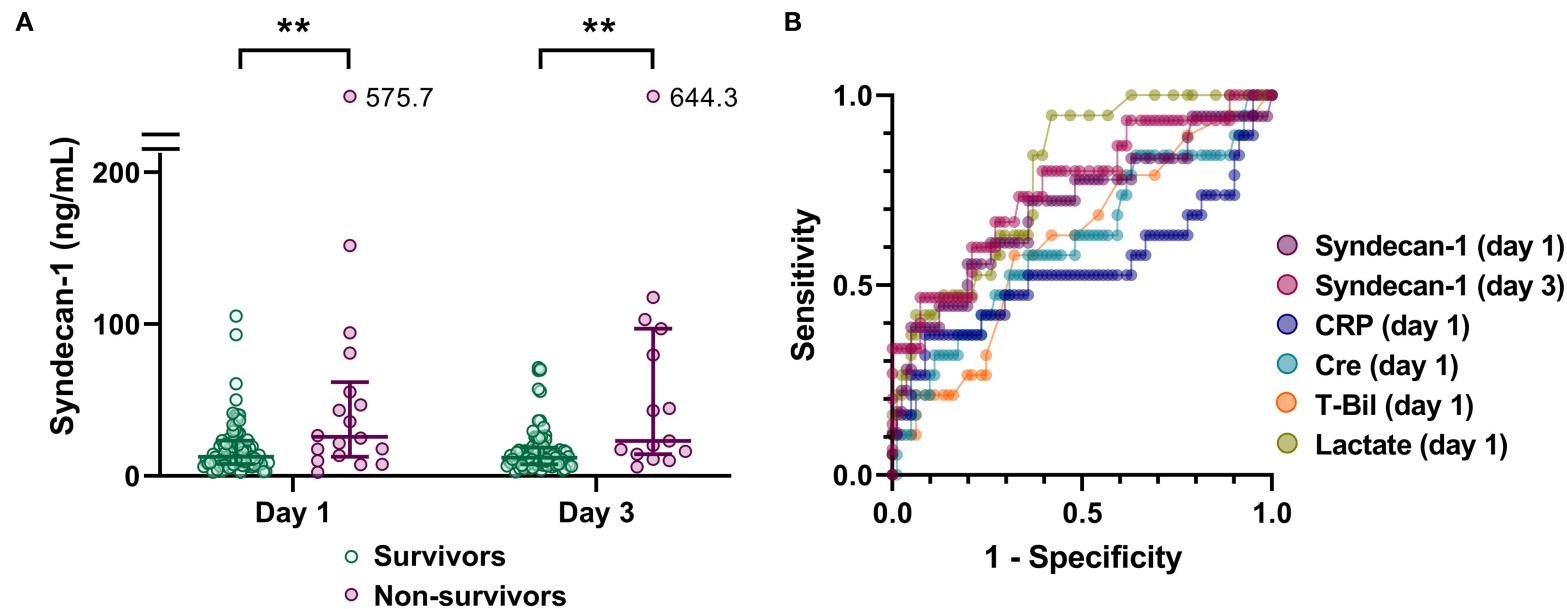
Circulating syndecan-1 is associated with fatal outcome. **(A)** Serum syndecan-1 levels in survivors and non-survivors (*n* = 81 and 18 on Day 1 and *n* = 81 and 15 on Day 3, respectively). The lines in the graph indicate the medians with interquartile ranges. The differences between the survivors and non-survivors were analyzed using the Mann–Whitney *U*-test. **P < 0.01. **(B)** Receiver-operating characteristics (ROC) curves for prediction of 28-day mortality are shown with respect to the following variables: syndecan-1 on Day 1, syndecan-1 on Day 3, C-reactive protein (CRP) on Day 1, creatinine (Cre) on Day 1, total bilirubin (T-Bil) on Day 1, and lactate on Day 1.

To investigate the potential utility of evaluating serum syndecan-1 levels in patients with suspected sepsis, we first analyzed the relationship between serum syndecan-1 and fatal outcome (Figure 2). Serum syndecan-1 levels in non-survivors were significantly higher than those in survivors on Day 1 (25.7 [13.5–55.4] vs. 12.7 [7.8–23.2] ng/mL, *P* < 0.01) and Day 3 (23.1 [15.3–88.4] vs. 12.0 [7.8–18.3] ng/mL, *P* < 0.01). Serum syndecan-1 levels were not significantly changed between Day 1 and Day 3 (Supplementary Figure 1) in both survivors (12.7 [7.8–23.2] vs. 12.0 [7.8–18.3] ng/mL, *P* = 0.13) and non-survivors (25.7 [13.5–55.4] vs. 23.1 [15.3–88.4] ng/mL, *P* = 0.61). In the ROC analysis for prediction of 28-day mortality, the AUCs were 0.704 for syndecan-1 on Day 1, 0.756 for syndecan-1 on Day 3, 0.612 for creatinine on Day 1, 0.615 for total bilirubin on Day 1, 0.543 for C-reactive protein on Day 1, and 0.791 for lactate on Day 1, suggesting that the prognostic value for syndecan-1 was higher than that of those for other markers of organ failure, but lower than that for tissue hypoxia.

### Circulating Syndecan-1 Is Associated With Organ Failure and Coagulation Failure

**Figure 3:**
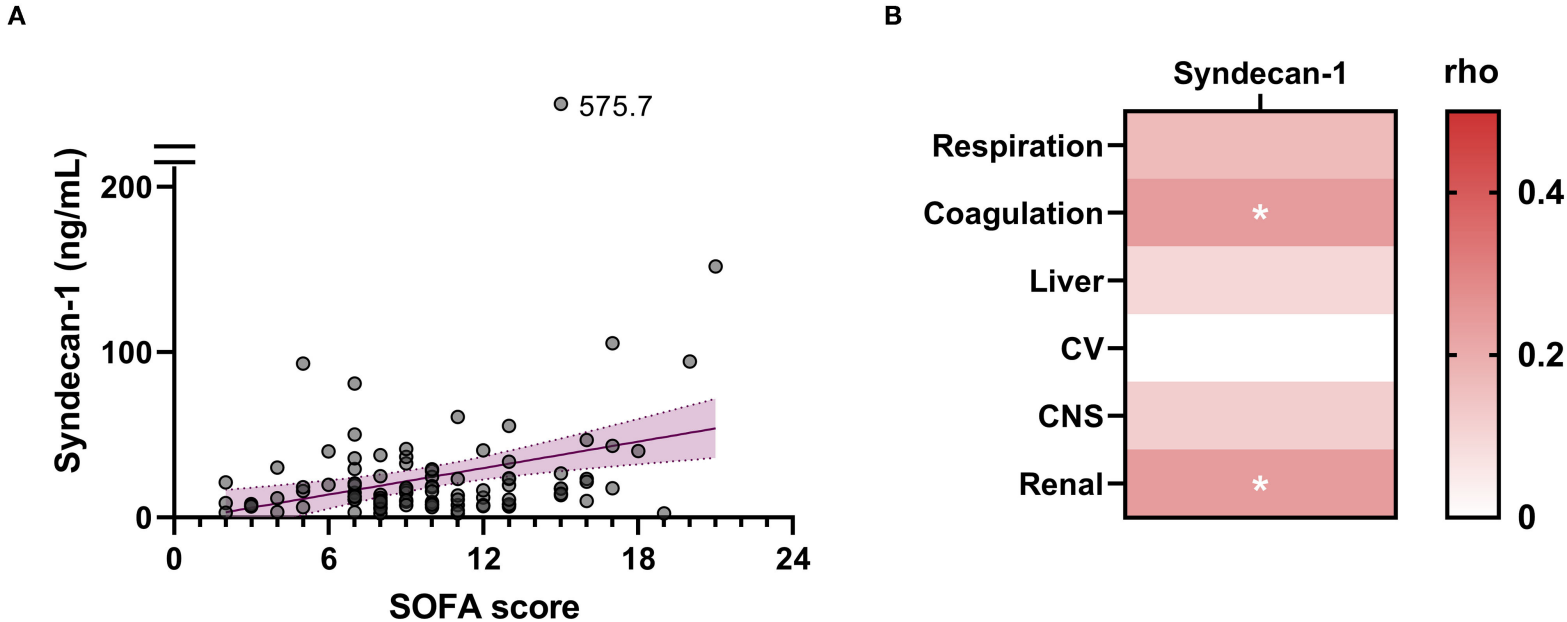
Circulating syndecan-1 is associated with multiple organ failure. **(A)** Relationship between serum syndecan-1 on Day 1 and SOFA score on Day 1. Spearman's rank correlation test indicated a weak but significant correlation (Spearman's rho = 0.23, *P* < 0.05). **(B)** Relationships between serum syndecan-1 on Day 1 and individual components of the SOFA score on Day 1. The intensities of the correlations are color-coded according to the Spearman's rho values. CV, cardiovascular system; CNS, central nervous system. *P < 0.05.

The higher SOFA scores observed in non-survivors compared with survivors (Table 1) indicated that multiple organ failure may be associated with fatal outcome. Spearman's rank correlation test indicated that serum syndecan-1 was weakly but significantly correlated with SOFA score (rho = 0.23, *P* < 0.05). Among the components of the SOFA score, coagulation score (rho = 0.24, *P* < 0.05) and renal score (rho = 0.24, *P* < 0.05) were significantly correlated with serum syndecan-1 (Figure 3).

**Figure 4:**
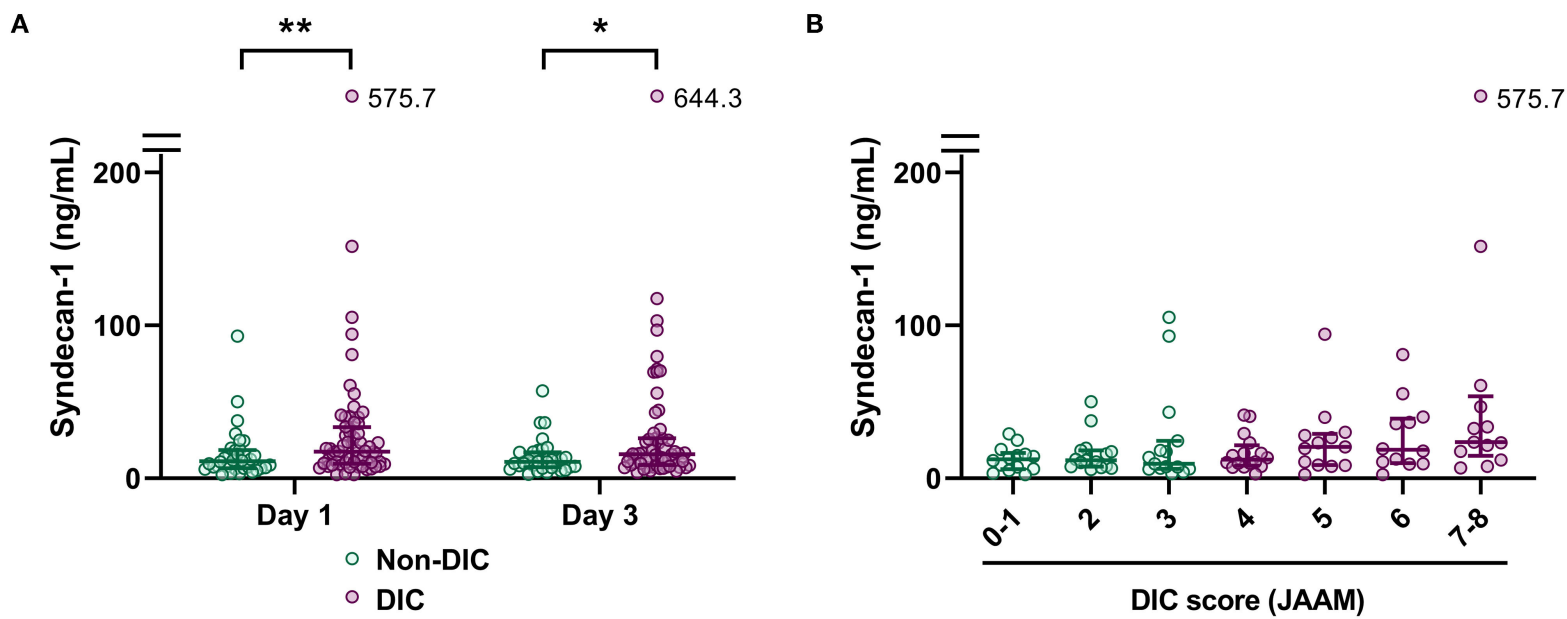
Circulating syndecan-1 is associated with disseminated intravascular coagulation (DIC). **(A)** Serum syndecan-1 levels in non-DIC and DIC patients (*n* = 34 and 64 on Day 1 and *n* = 33 and 62 on Day 3, respectively). The lines in the graph indicate the medians with interquartile ranges. The differences between non-DIC and DIC patients were analyzed using the Mann–Whitney *U*-test. *P < 0.05. **P < 0.01. **(B)** Relationship between serum syndecan-1 on Day 1 and DIC score on Day 1. Spearman's rank correlation test indicated a weak but significant correlation (Spearman's rho = 0.33, *P* < 0.01).

To further investigate the relationship between serum syndecan-1 and coagulation failure, we compared the serum syndecan-1 levels in non-DIC and DIC patients. Among the 100 patients with sepsis in this study, 65 fulfilled the JAAM criteria for DIC during the first week of ICU stay. As shown in Figure 4, the serum syndecan-1 levels on Day 1 and Day 3 were significantly higher in DIC patients compared with non-DIC patients (Day 1: 17.5 [9.7–33.2] vs. 11.2 [6.6–18.3] ng/mL, *P* < 0.01; Day 3: 15.7 [8.6–26.0] vs. 10.8 [7.4–16.9] ng/mL, *P* < 0.05). Spearman's rank correlation test indicated that serum syndecan-1 was weakly but significantly correlated with DIC score (rho = 0.33, *P* < 0.01).

### Circulating Syndecan-1 Is Associated With Persistent Thrombocytopenia

**Figure 5:**
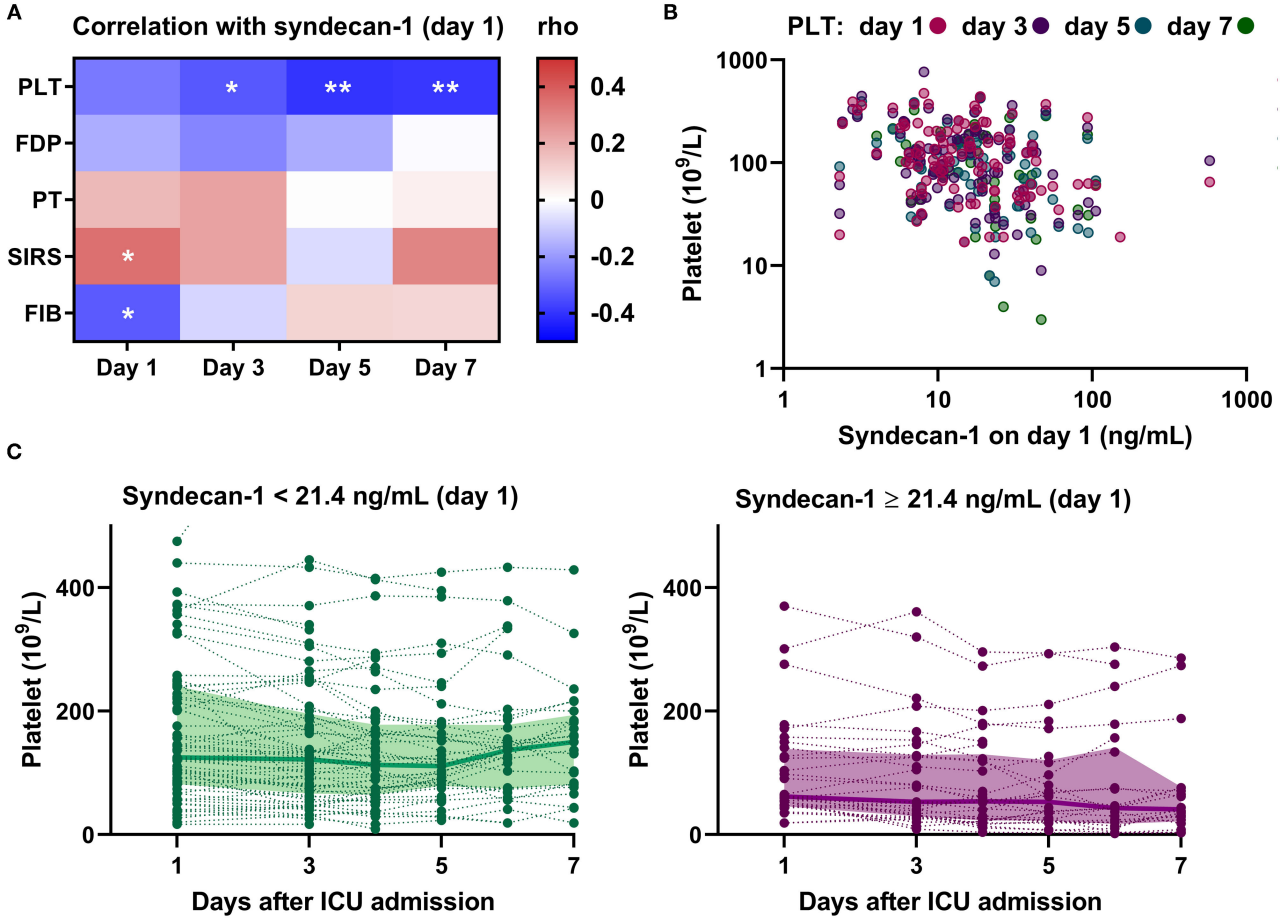
Circulating syndecan-1 is associated with persistent thrombocytopenia. **(A)** Relationships between serum syndecan-1 on Day 1 and individual components of the DIC score on Days 1, 3, 5, and 7 in the subpopulation who stayed in the ICU until at least Day 7 (*n* = 43). The intensities of the correlation are color-coded according to the Spearman's rho values, with red and blue colors indicating positive and negative correlations, respectively. PLT, platelets; FDP, fibrin(ogen) degradation products; PT, prothrombin time; SIRS, systemic inflammatory response syndrome score; FIB, fibrinogen. *P < 0.05. **P < 0.01. **(B)** Correlations between serum syndecan-1 on Day 1 and platelet counts on Days 1, 3, 5, and 7 in the whole population with sepsis (*n* = 99 on Day 1; *n* = 97 on Day 3; *n* = 69 on Day 5; *n* = 43 on Day 7). **(C)** Platelet counts on Days 1, 3, 4, 5, 6, and 7 in patients with low serum syndecan-1 (<21.4 ng/mL, *n* = 67) and high serum syndecan-1 (≥21.4 ng/mL, *n* = 32). The bold lines indicate the median values and the filled-in areas indicate interquartile ranges. The optimal cut-off value (21.4 ng/mL) was determined by ROC analysis (Supplementary Figure 2).

Next, we analyzed the correlations between serum syndecan-1 on Day 1 and individual components in the DIC criteria, such as platelet count and PT, on Days 1, 3, 5, and 7 (Figures 5A,B). For this, we analyzed the subpopulation who stayed in the ICU until at least Day 7 (*n* = 43). Interestingly, serum syndecan-1 on Day 1 was more strongly associated with platelet count on Day 5 (rho = −0.40, *P* < 0.01) or Day 7 (rho = −0.39, *P* < 0.01) than with platelet count on Day 1 (rho = −0.26, *P* = 0.09) or Day 3 (rho = −0.33, *P* < 0.05). Indeed, patients with high serum syndecan-1 showed delayed recovery from thrombocytopenia (Figure 5C). These findings indicate that circulating syndecan-1 may predict platelet responses to injured vascular walls over the next few days.

## Discussion

In the present study, we have shown that elevated circulating syndecan-1 on the first day of ICU admission may predict persistent thrombocytopenia and lethal outcome in patients with suspected sepsis. Thrombocytopenia is a frequent complication of sepsis, and is associated with impaired vascular integrity, organ failure, and poor outcome (16). It is not necessarily linked with activation of intravascular coagulation, but is related to activation of complement signaling. In general, platelet counts in patients with sepsis remain low during the first 4–5 days after ICU admission and then increase over time, especially in less severe cases (16). However, severe cases remain thrombocytopenic for prolonged periods of ICU stay. The present findings indicate that circulating syndecan-1 levels can discriminate these two subpopulations at the time of ICU admission. Impaired vascular integrity may be the underlying mechanism linking elevated serum syndecan-1 and persistent thrombocytopenia (Figure 1).

Our findings are consistent with previous studies showing that syndecan-1 levels were associated with coagulation failure in patients with sepsis (11, 12, 17). Specifically, Ikeda et al. showed that syndecan-1 levels on day 1 negatively correlated with fibrinogen levels and platelet counts in patients with sepsis (11). Piotti et al. showed that syndecan-1 levels on day 1 predicted incident coagulation failure, which was defined as platelet counts of <50 × 10^9^/L (12). Ostrowski et al. showed that syndecan-1 levels correlated with hypocoagulability assessed by thrombelastography (17). These findings indicate that elevated circulating syndecan-1 is associated with consumption coagulopathy rather than intravascular activation of coagulation.

Besides coagulation failure, renal dysfunction was associated with elevated circulating syndecan-1 (Figure 3B). This is consistent with previous studies showing that elevated syndecan-1 levels were associated with higher incidence of acute kidney injury in patients with severe trauma or sepsis (18–20). Furthermore, a recent study indicated that changes in kidney function alone can induce several-fold changes in circulating syndecan-1 (21). An acute reduction in renal blood flow and/or glomerular filtration rate results in a marked reduction in renal clearance of syndecan-1 and a marked elevation in serum syndecan-1. This should be taken into account while assessing serum syndecan-1 levels as an indicator of glycocalyx degradation.

The present study has several limitations. First, the limited number of patients in this single-center study did not allow us to conduct multivariate analysis. Thus, the influence of confounding factors was undetermined. Second, patients who were discharged from the ICU were not followed up for the study. Accordingly, 81 survivors and 19 non-survivors were analyzed on Day 1, while 32 survivors and 12 non-survivors were analyzed on Day 7. This may distort the composition of the subjects. Third, the diagnosis of sepsis was not confirmed in all patients in this study because the inclusion criteria were patients with suspected infections who required intensive care. All patients had a SOFA score of ≥2 on Day 1, but the SOFA scores before ICU admission were not available in some patients. Further studies are needed to elucidate the potential utility of measuring serum syndecan-1 levels in patients with sepsis.

## Conclusions

Elevated circulating syndecan-1 on the first day of ICU admission was associated with persistent thrombocytopenia and lethal outcome in patients with suspected sepsis.

## Data Availability Statement

The raw data supporting the conclusions of this article will be made available by the authors, without undue reservation.

## Ethics Statement

This study was approved by the Ethics Committee of Kagoshima University Graduate School of Medical and Dental Sciences, Kagoshima, Japan (210026). Written informed consent was obtained from all patients for research use of their leftover blood samples after routine blood tests.

## Author Contributions

KH analyzed the clinical data. TI designed the experimental protocol and wrote the manuscript. SY participated in the laboratory experiments. YM, CK, SN, IM, and YK critically appraised the manuscript. All authors have read and approved the final manuscript.

## Funding

This work was supported in part by a research grant from the Japan Society for the Promotion of Science (Grant-in-Aid 21H03033).

## Conflict of Interest

The ELISA for syndecan-1 is a product in development of Shino-Test Corporation, where SY is an employee. IM holds endowed faculty positions at Kagoshima University and receives research funding from Shino-Test Corporation. The funding is for academic promotion and is not directly related to the present study. The remaining authors declare that the research was conducted in the absence of any commercial or financial relationships that could be construed as a potential conflict of interest.

## Publisher's Note

All claims expressed in this article are solely those of the authors and do not necessarily represent those of their affiliated organizations, or those of the publisher, the editors and the reviewers. Any product that may be evaluated in this article, or claim that may be made by its manufacturer, is not guaranteed or endorsed by the publisher.
