# Supplementary figures and images for: Circulating Syndecan-1 as a Predictor of Persistent Thrombocytopenia and Lethal Outcome: A Population Study of Patients With Suspected Sepsis Requiring Intensive Care
Source: Front Cardiovasc Med. 2021 Sep 7;8:730553. doi: 10.3389/fcvm.2021.730553 (PMC8452900; doi:10.3389/fcvm.2021.730553)

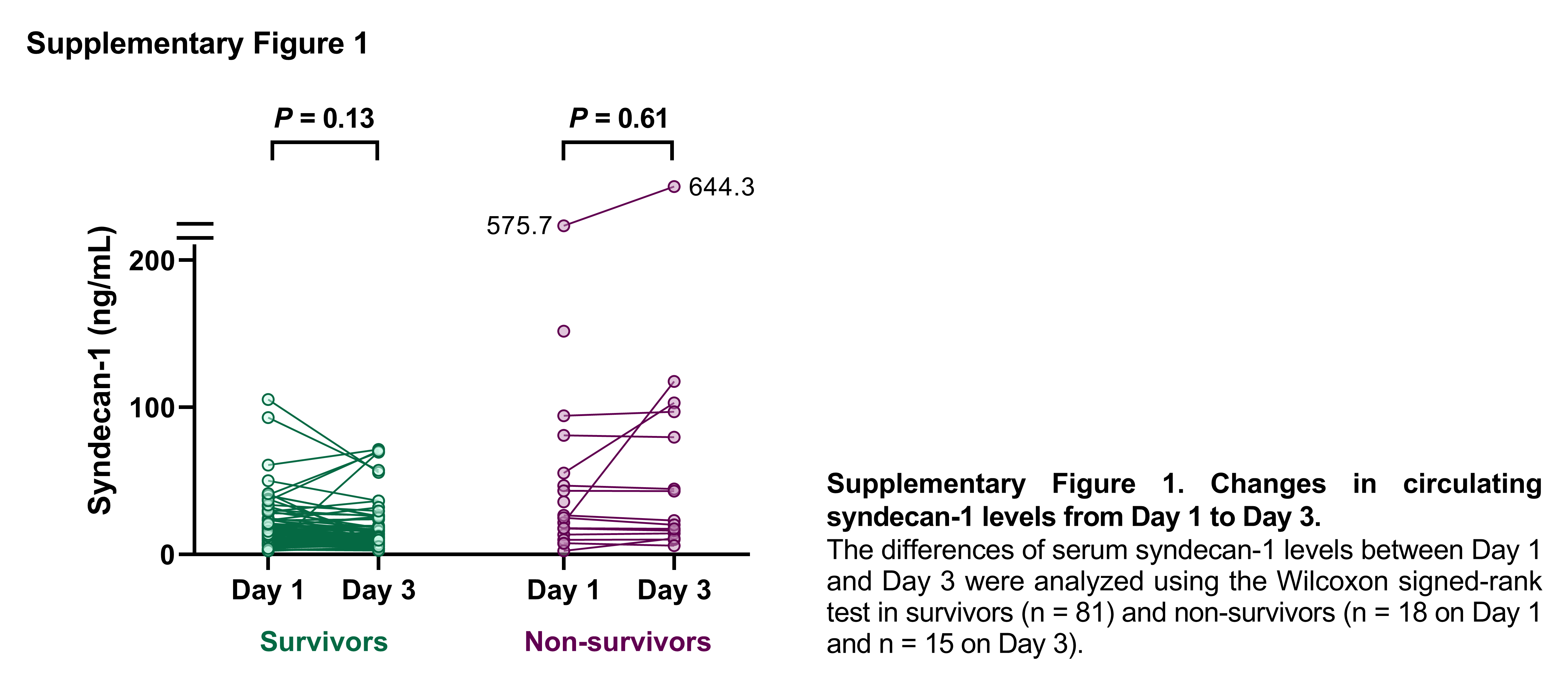

Supplement: Supplementary file 1 [file Image_1.TIF]

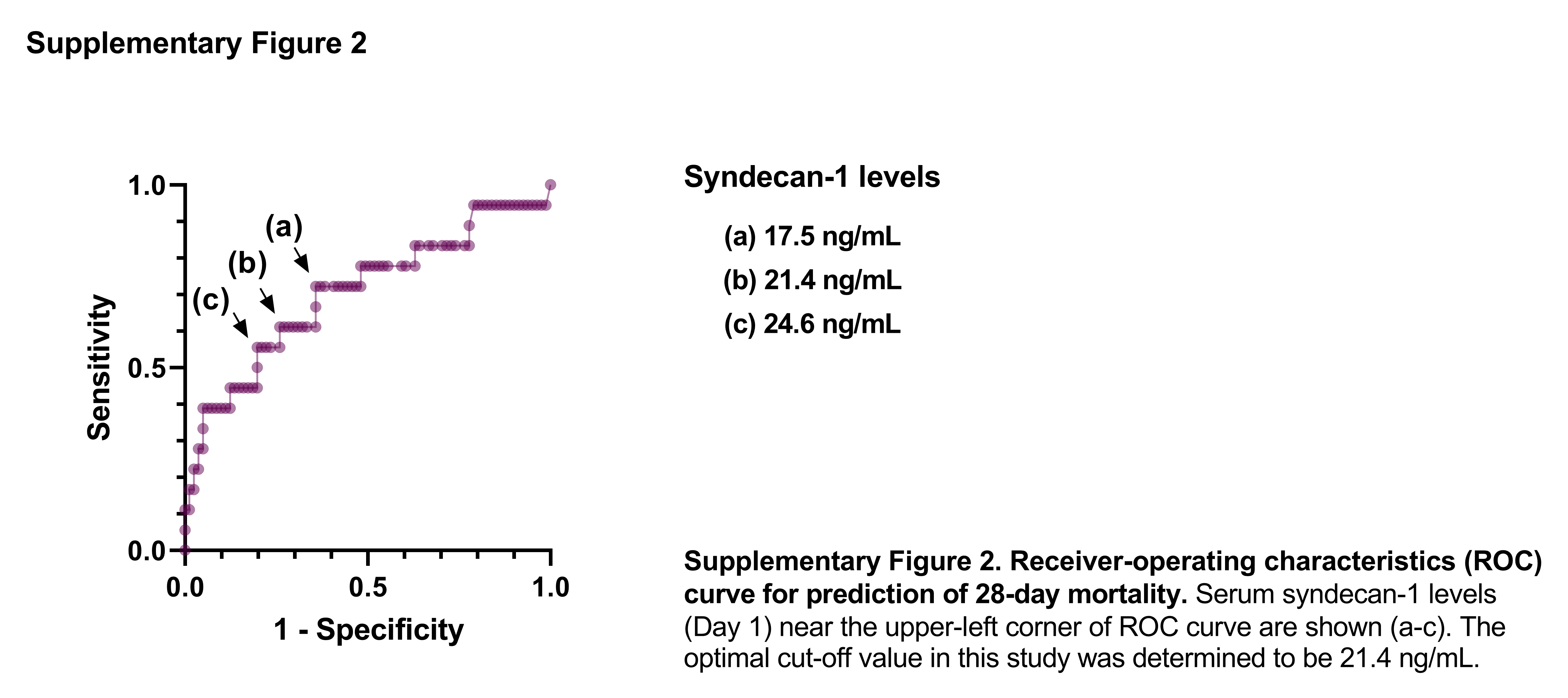

Supplement: Supplementary file 2 [file Image_2.TIF]
